# Less Is More: Adaptive Trainable Gradient Dropout for Deep Neural Networks

**DOI:** 10.3390/s23031325

**Published:** 2023-01-24

**Authors:** Christos Avgerinos, Nicholas Vretos, Petros Daras

**Affiliations:** Information Technologies Institute (ITI), Centre for Research and Technology Hellas (CERTH), 57001 Thessaloniki, Greece

**Keywords:** adaptive dropout, gradient dropout, gradient freezing, trainable dropout

## Abstract

The undeniable computational power of artificial neural networks has granted the scientific community the ability to exploit the available data in ways previously inconceivable. However, deep neural networks require an overwhelming quantity of data in order to interpret the underlying connections between them, and therefore, be able to complete the specific task that they have been assigned to. Feeding a deep neural network with vast amounts of data usually ensures efficiency, but may, however, harm the network’s ability to generalize. To tackle this, numerous regularization techniques have been proposed, with dropout being one of the most dominant. This paper proposes a selective gradient dropout method, which, instead of relying on dropping random weights, learns to freeze the training process of specific connections, thereby increasing the overall network’s sparsity in an adaptive manner, by driving it to utilize more salient weights. The experimental results show that the produced sparse network outperforms the baseline on numerous image classification datasets, and additionally, the yielded results occurred after significantly less training epochs.

## 1. Introduction

In recent years, artificial neural networks have demonstrated undeniable efficiency in carrying out tasks such as image detection, action recognition, and compression, rendering their implementations almost exclusive candidates for problem solving in such domains. In conjunction with the technological advancements in computational power and data handling, faster and more accurate networks are constantly designed, based on dense, recursive architectures that are able to analyze more complex data. The efficiency of deeper networks lies in the fact that these networks feature significantly more trainable parameters than shallow networks, making them extremely flexible in interpreting diverse input data. Although this is a major advantage, the excessive adaptation of a network’s neurons and synapses to the available data establishes a risk of overfitting, and thus can render the model unable to generalize.

In order to exploit the performances of very complex networks and concurrently ameliorate this inherit adversity, several regularization techniques have been proposed, such as cross validation [1,2], bagging [3], boosting weights [4], data augmentation [5,6,7], early stopping [8], and weight decay [9]. One of the most efficient, effective, and therefore popular regularization techniques is dropout [10], which tackles overfitting by randomly removing nodes during training, with no additional computational overhead. Dropout benefits the network by randomly adding noise to its hidden units, forcing the loss descent path to frequently change and avoid settling to a local minimum. Dropout essentially prevents the co-adaptation of activations, so that a network’s hidden units detect features independently of each other. Although this method usually ensures better model performance, dropout comes at the cost of a far more important convergence time. Randomly changing the network’s loss descent path can multiply the necessary training time, as the model seeks for a general representation that fits the given input, using varying parts of its components at each iteration. More specifically, for a network with *t* parameters, if all of them are considered eligible for dropout, the number of possible loss descent paths would be $2^{t}$.

This paper proposes a learnable adaptive training method that aims at producing efficient models that are able to generalize accurately and fast. Extending the standard random dropout technique, the proposed method freezes a part of the network’s parameters based on the input information on every training step by zeroing targeted gradients. The selection of gradients to be switched off is carried out by an ensemble of networks with trainable parameters. The proposed method was evaluated by conducting experiments on popular public image classification datasets, showing that a simple vanilla network with dropout is outperformed by its modified learned sparse version.

The incentive behind the presented work is to enhance the standard behavior of the dropout mechanism. The proposed method achieves this by selecting which weights and gradients are frozen in each training step, instead of ambiguously dropping potentially important ingredients of the network, i.e., the essence of the vanilla dropout technique.

The remainder of this paper is organized in four sections: Section 2 is a presentation of prior publications on relative scientific domains. In Section 3, the description of the idea and implementation of the presented method are detailed. Section 4 is an ablation study on the performed experiments, and additionally analyzes the yielded results. Finally, Section 5 concludes the paper.

## 2. Related Work

Recently, numerous works have focused on tackling the overfitting problem that occurs when complex artificial networks are implemented. Variations and extensions of the dropout mechanism have been proposed, such as DropConnect [11], which inducts network sparsity by randomly dropping the weights of a fully connected layer, instead of its activations’ output vectors. In [12], the authors propose a regularizing method that, when applied to very complex residual networks, randomly drops fractions of layers. This approach aims at sparsifying the network during training, while retaining its complexity during test time and thus boosting generalization while retaining performance. DropBlock [13] is a generalization of Cutout [14], a data augmentation method where random square regions of an image are masked out to improve performance and robustness on object occlusion examples. In DropBlock, the authors apply this method to the feature maps of convolution filters, aiming at better generalization of such layers with spatial structure.

Other papers aim to improve the generalization ability of multi-branch networks by blocking possible co-adaptation between parallel branches or paths, either by randomly dropping network fractions, as in [15,16], or by modifying activation functions, as in [17,18]. In other works, the dropout mechanism is extended, such as in Maxout [19], which introduces a new layer that essentially generalizes the rectified linear unit (ReLU) and leaky ReLU functions and exploits the averaging properties of dropout. In [20], instead of masking out weights, a random gradient regularization mechanism is introduced, inducing noise to gradients in order to improve model generalization.

All mentioned dropout variations are established on randomly infusing noise to either weights, activations, or gradients. During training, the weight, activity, or gradient of a hidden unit is set to zero with a fixed probability using samples from a Bernoulli distribution. On the contrary, approaches such as [21] are closely related to the presented work, as the probability of a unit being dropped is not random but strongly related to the candidate unit’s inputs and activation. A similar intuition was followed in [22], where units and weights were ranked using an approximate rank of importance. The ambition of that paper was to reduce the dependency between the important and unimportant features of a network, i.e., to maximize the mutual information between units in the same layer, so that the impact of dropping a unit is minimal. In [23], the authors proposed a learning-rate dropout mechanism similar to the original dropout approach; upon each step, a random unit’s learning rate drops to zero, thereby temporarily disabling its training. Although the main concept of that work is closely related to the present paper’s idea, they differ on the important aspect of randomly freezing the network’s nodes, instead of selectively masking the unimportant units.

## 3. Proposed Method

**Figure 1:**
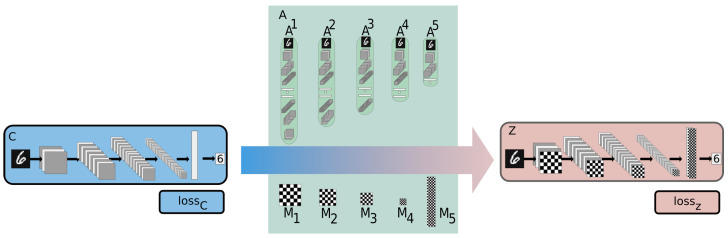
Example of the proposed method. The auxiliary network ensemble **A**, comprising networks $A^{1},A^{2},\ldots ,A^{k},k\in 0,1,2,\ldots ,L^{C}$, is responsible for providing each layer of network *C* with a binary mask $M_{k}$, which controls which parts of the $l_{j}^{C}$ layer will be trained. The applied masks’ performance is evaluated on the next forward pass, and, if the proposals are accepted, the training procedure continues with the modified version of *C*, *Z*.

The proposed approach is an end-to-end trainable architecture as can be seen in Figure 1, established on a continuous three-way communication channel between its components—more specifically, a core network *C* with its set of parameters (weights and biases) denoted as $\theta^{C}$, a modified network Z with $\theta^{Z}$, and a set of auxiliary networks, A, comprising networks $A^{1},A^{2},\ldots ,A^{k},k\in 0,1,2,\ldots ,L^{C}$.

We index the layers of a network *U* as $l_{j}^{U},j\in 1,2,\ldots ,L^{U}$, with $L^{U}$ representing the total number of layers in a network *U*. Although each network is independently trained, back propagated, and optimized for a specific objective, the information exchanged between them is crucial for the maximal exploitation of the salient weights of network *C*.

On each training step, *C* represents a deep neural network that aims to minimize the cost function:

(1) $$J=-\frac{1}{T}\left(\sum_{i=1}^{T}y_{i}\cdot \log\left({\hat{y}}_{i}\right)\right),i\in 1,2,\ldots ,T,$$

where *i* is the input, *T* is the cardinality of the training dataset, $y_{i}$ is the ground truth value, and ${\hat{y}}_{i}$ the model’s estimation.

**Figure 2:**
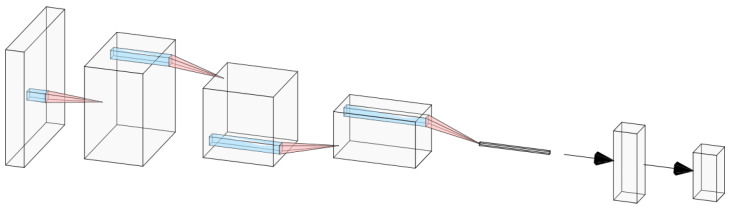
Architecture of the *C* network consisting of the input image, four convolutional layers, and two fully connected layers. The proposed method is beneficial to the network even in such minimal setups.

**Figure 3:**
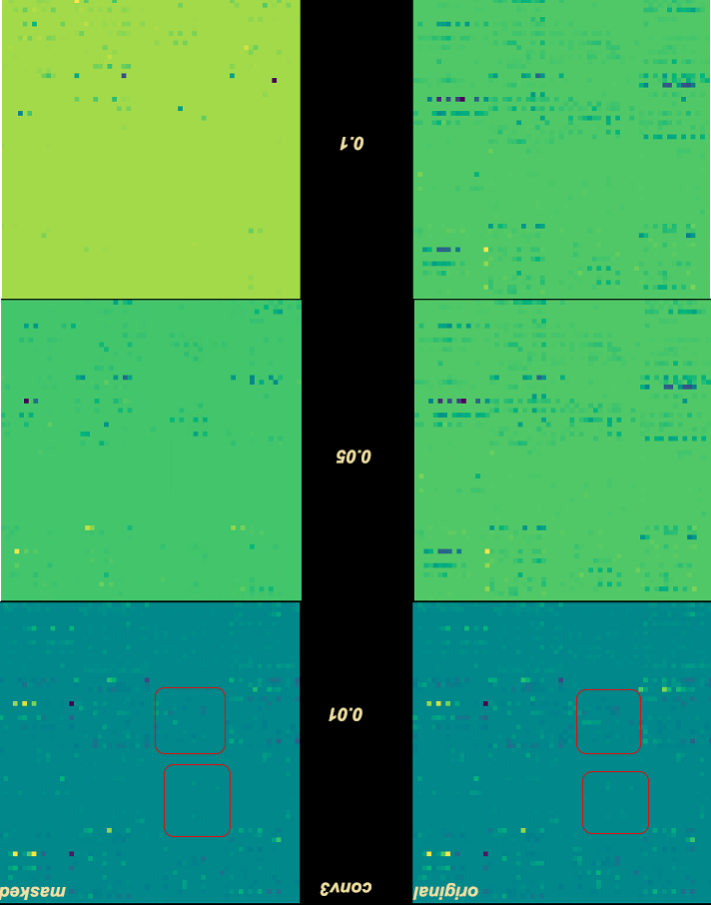
Threshold effect on the third convolution filter, for p=0.01, p=0.05, and p=0.1. Red squares in the first dense example indicate same pixel neighborhoods for easier comprehension.

The ensemble of auxiliary networks, A, is responsible for providing *C* (Figure 2) with a set of binary masks, B. In order to bolster the usage of salient features for each layer of *C*, the proposed method implements a convolutional variational autoencoder network for each auxiliary subnetwork, $A^{k}$. More information on the architecture of each network can be found in Section 4. First, $l_{j}^{C}$’s weights are encoded to a lower dimension by the respective encoder of $A^{k}$, and then, the decoder side attempts to reproduce them while trying to ignore less important weights. The generated representations are the blueprint from which the desired binary masks are extracted. The binary value, $m_{j}^{n}$, of a neuron *n* contained in layer $l_{j}^{A^{k}}$ is decided by taking into account the neuron’s absolute gradient value, $|g_{j,n}^{A^{k}}|$, after back propagation, compared to the strongest absolute gradient value per layer, so that

(2) $$m_{j}^{n}=\left\{\begin{array}{cc} 1 & \;\mathrm{if}\;\left|g_{j,n}^{A^{k}}\right|>p_{j}\cdot {max}_{\left|G_{j}^{A^{k}}\right|}\\ 0 & \;\mathrm{else}\; \end{array}\right\},n\in 1,2,\ldots ,\left|l_{j}^{A^{k}}\right|$$

where $p_{j}$ represents a masking threshold $0\leq p_{j}\leq 1$, $j\in 1,2,\ldots ,L^{K}$ for each layer of *C*, and $G_{j}^{A^{k}}$ represents the gradient matrix of layer $l_{j}^{A^{k}}$. A higher masking threshold $p_{j}$ implies that the masking mechanism will be more aggressive towards that specific layer. The effects of the threshold can be seen in Figure 3.

Apart from acquiring masks from a layer’s gradients, and in a similar manner, the method takes into consideration the absolute weight value of each neuron, $|w_{j,n}^{A^{k}}|$, after some training intervals, so that:

(3) $$m_{j}^{n}=\left\{\begin{array}{cc} 1 & \;\mathrm{if}\;\left|w_{j,n}^{A^{k}}\right|>p_{j}\cdot {max}_{\left|W_{j}^{A^{k}}\right|}\\ 0 & \;\mathrm{else}\; \end{array}\right\},n\in 1,2,\ldots ,\left|l_{j}^{A^{k}}\right|$$

where $W_{j}^{A^{k}}$ represents the weight matrix of layer $l_{j}^{A^{k}}$.

Filtering parts of a layer $l_{j}^{C}$ by the respective $l_{j}^{A^{k}}$ weight values provides more robust mask proposals and acts as a review of the freezing procedure thus far. After some training epochs, the network has built an effective generalization mechanism with more stable weights than before, the values of which quantify their respective participation levels in minimizing the loss function (1).

The assigned binary values $m_{j}^{n}$ of all neurons in layer $l_{j}^{A^{k}}$ constitute the binary mask $M_{j}\in B$. Each produced binary mask corresponds to each *C* layer, $l_{j}^{C}$, and controls how different parts of that layer are trained, by computing the Hadamard product between the individual values of the mask and the respective layer’s gradient values, $G_{j}^{C}$, after the latter have been obtained through back propagation. The Hadamard product is calculated as:

(4) $$H_{j}=M_{j}⊙G_{j}^{C}=\left(\begin{array}{ccc} {M_{j}}^{11}\cdot {G_{j}^{C}}^{11} & \ldots & {M_{j}}^{1N_{j}^{C}}\cdot {G_{j}^{C}}^{1N_{j}^{C}}\\ \vdots & \ddots & \vdots \\ {M_{j}}^{N_{j}^{C}1}\cdot {G_{j}^{C}}^{N_{j}^{C}1} & \ldots & {M_{j}}^{N_{j}^{C}N_{j}^{C}}\cdot {G_{j}^{C}}^{N_{j}^{C}N_{j}^{C}} \end{array}\right)$$

where ⊙ denotes the Hadamard product of two matrices, and $N_{j}^{C}$ the size of each dimension of a $l_{j}^{C}$ layer.

The gradients computed on every training step express the magnitudes of adjustments that network *C* needs to apply at each neuron so that Function (1) is minimized. The concept of freezing neurons that only need minor adjustments during training aims at a faster convergence time, as the network is targeted towards modifying the weights of neurons that need these adjustments the most.

The effectiveness of the applied masks is evaluated on the next forward pass of *C*. More specifically, on each training step, the input data are propagated through *C*, and through its modified counterpart, *Z*, which contains the updated weights, based on the masked gradients, $H_{j}$, of the previous step. Depending on the calculated loss for each network, $loss_{C}$ and $loss_{Z}$, the algorithm either accepts the proposed parameters after masking, $\theta^{Z}$, consisting of all modified gradients $G^{Z}$, and weights $W^{Z}$, or rejects them and advances with the original parameters $\theta^{C}$, i.e., the unmasked gradients $G^{C}$ and weights $W^{C}$, as updated by *C*’s backward pass. Finally, either $loss_{C}$ or $loss_{Z}$ is passed to the auxiliary networks, $A^{1},A^{2},\ldots ,A^{L^{C}}$.

Although each auxiliary network $A^{k}$ is an unsupervised generative model, its weights are updated by inheriting the loss value of a supervised classification neural network, $loss_{C}$ or $loss_{Z}$. This behavior proves that the method is agnostic towards the individual networks’ architectures, and shows that the intertwined behavior of the components ensures uninterrupted, end-to-end integration.

## 4. Experiments and Results

The proposed method consists of an ensemble of networks, and its implementation can become cumbersome when deeper architectures are employed. Testing the method on upscaled networks is out of the scope of the proposed paper, as all state of the art methods for dropout are tested against their respective vanilla dropout versions. The experimental results show that a typical convolutional neural network is benefited when utilizing the proposed adaptive dropout method, when compared to a standard dropout integration.

### 4.1. Datasets

- **CIFAR-10** [24]: The CIFAR-10 dataset features 60,000 32 × 32 color images, divided into 10 classes of 6000 images each. The training set consists of 50,000 images, whereas the test set contains 10,000 images, randomly selected from each class.
- **USPS Handwritten Digits (USPS)** [25]: USPS is a dataset of handwritten digits featuring 7291 training and 2007 8 × 8 testing examples, coming from 10 classes.
- **Fashion-MNIST** [26]: Fashion-MNIST is structured based on MNIST [27], a handwritten digit dataset, which is considered an almost solved problem, and is designed as a more challenging dataset; it consists of clothing images divided into a training set of 60,000 samples and a test set of 10,000 28 × 28 grayscale samples of 10 classes.
- **SVHN** [28]: SVHN is an image dataset of house numbers, obtained from Google Street View images. The dataset’s structure is similar to that of the MNIST dataset; each of the 10 classes consists of images of one digit. The dataset contains over 600,000 digit images, split into 73,257 digits for training, 26,032 digits for testing, and 531,131 additional training examples.
- **STL-10** [29]: The STL-10 dataset is an image recognition dataset inspired by the CIFAR-10 dataset. The dataset shares the same structure as the CIFAR-10 dataset, with 10 classes of 500 96 × 96 training images and 800 96 × 96 test images in each. However, the dataset also contains 100,000 unlabeled images for unsupervised training, with content extracted from similar, but not the same categories as the original classes, acquired from Imagenet [30]. Although this dataset was designed for developing scalable unsupervised methods, in this study, it was used as a standard supervised classification dataset.

### 4.2. Implementation Details

The core network *C* consists of four convolutional layers, each activated by a leaky ReLU function and normalized by a batch normalization layer. The resulting feature map is then flattened and fed to a fully connected layer, also activated by a leaky ReLU function and followed by a batch normalization layer. The vanilla version of *C* randomly drops some elements of the network by applying standard dropout with probability $p_{drop}=0.25$. The output is finally passed to a second fully connected layer and then activated by a multi-class softmax function.

For every $l_{j}^{C}$, an auxiliary network is built and trained in order to provide the required binary mask to that specific layer. The auxiliary networks for the proposed approach are based on variational autoencoders [31] with a fixed encoding section of four convolutional filters and a modified decoding part of transposed convolutions, their number depending on the size of the output mask to be applied on the $l_{j}^{C}$ gradients or weights. All filters are activated using leaky ReLU activation functions, as they are slightly faster than normal ReLU functions and alleviate the “dying ReLU” problem [32].

Variational autoencoders map their inputs to a distribution instead of a fixed feature map; then, using the mean vector μ and the standard deviation vector σ, a sample of the distribution can be fed to the decoding part of the network. Using the reparameterization trick, the output of the decoder is backpropagated through the network, training the μ and σ vectors, and also used for generating the essential binary masks, as described in the previous section.

For each experiment, the network was initialized by training in its vanilla version until it reached a minimum validation accuracy of about 30%, which was typically achieved in the first epoch. The model parameters were optimized using stochastic gradient descent, whereas the learning rate was initialized at 0.01 and decayed every 5 epochs by 0.5.

### 4.3. Results

**Figure 4:**
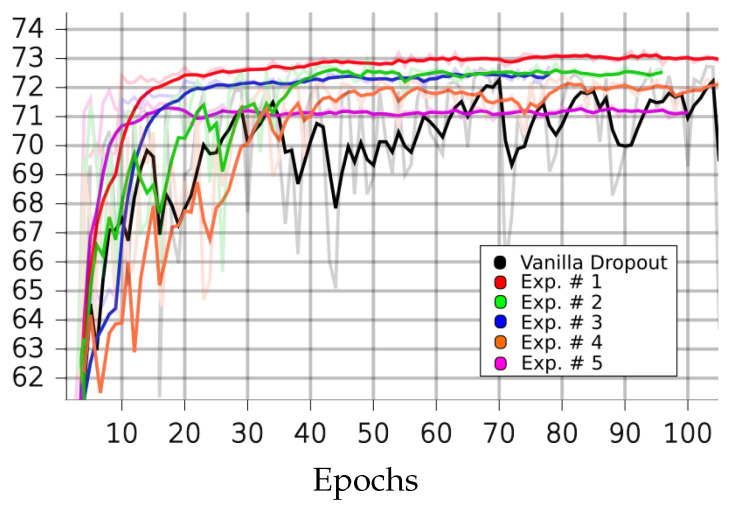
Performances of LIM and vanilla dropout methods on the CIFAR10 dataset, trained and tested for 100 epochs. Graphs were smoothed for better comprehension; original graphs can be seen in the background. Curves correspond to Table 2 scores. Black: vanilla. Red: 1. Green: 2. Blue: 3. Orange: 4. Purple: 5.

**Figure 5:**
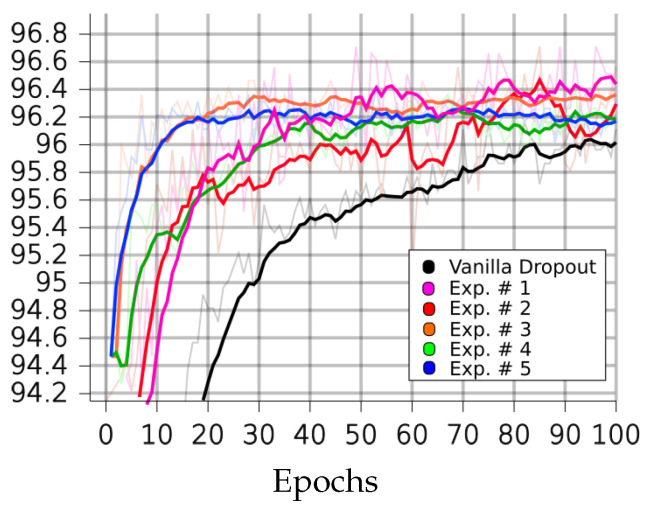
Performance of LIM and vanilla dropout methods on the USPS dataset, trained and tested for 100 epochs. Graphs were smoothed for better comprehension; original graphs can be seen in the background. Curves correspond to Table 2 scores. Black: vanilla, purple: 1, red: 2, orange: 3, green: 4, blue: 5.

**Figure 6:**
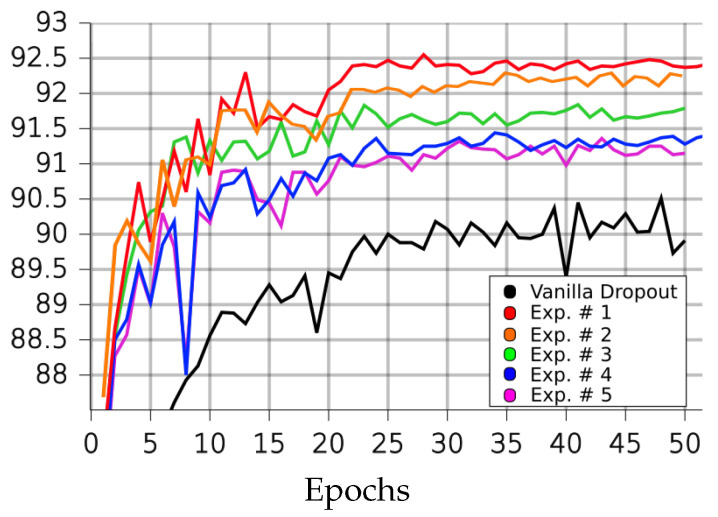
Performances of LIM and vanilla dropout methods on the fashion-MNIST dataset, trained and tested for 50 epochs. Curves correspond to Table 3 scores; black: vanilla, red: 1, orange: 2, green: 3, blue: 4, purple: 5.

**Figure 7:**
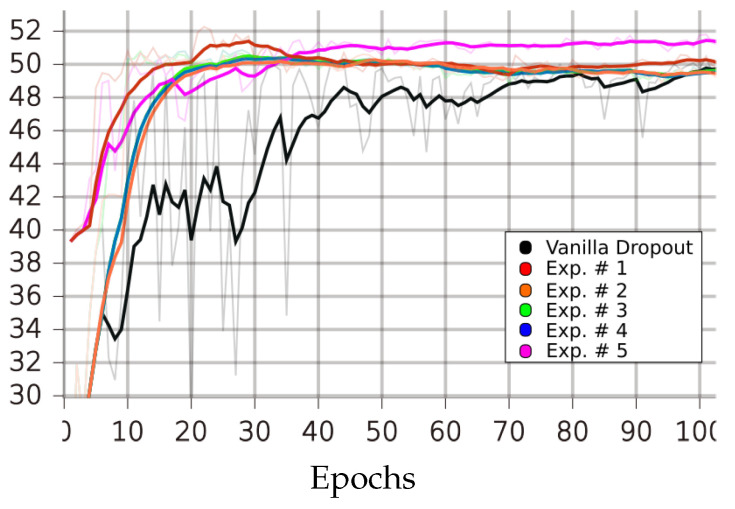
Performances of LIM and vanilla dropout methods on the STL-10 dataset, trained and tested for 100 epochs. Graphs were smoothed for better comprehension; original graphs can be seen in the background. Curves correspond to Table 4 scores; black: vanilla, red: 1, purple: 2, green: 3, orange: 4, blue: 5.

**Figure 8:**
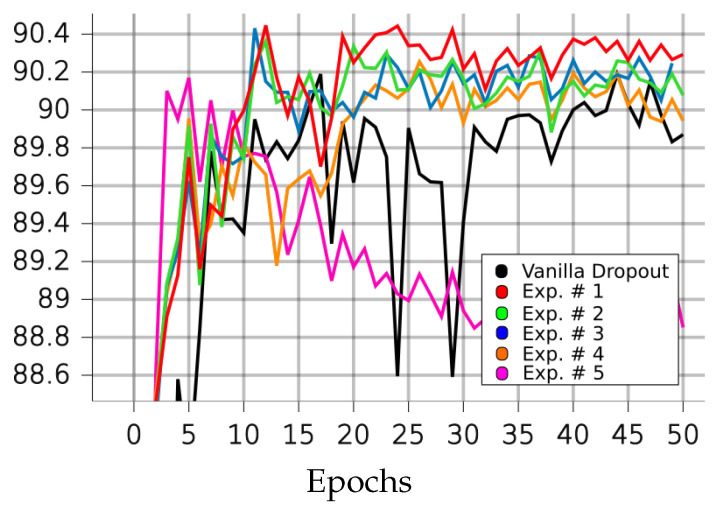
Performances of LIM and vanilla dropout methods on the SVHN dataset, trained and tested for 50 epochs. Graphs were smoothed for better comprehension; original graphs can be seen in the background. Curves correspond to Table 5 scores; black: vanilla, red: 1, green: 2, blue: 3, orange: 4, purple: 5.

Table 1, Table 2, Table 3 and Table 4 and Figure 4, Figure 5, Figure 6, Figure 7 and Figure 8 demonstrate how different setup parameters of the proposed method lead to either better accuracy or faster convergence time, when compared to the standard vanilla dropout. The first two columns of each table hold the accuracy score for each setup and the epochs at which they were performed, respectively. Columns 3 to 7 are the thresholds *p* used by the A network ensemble to acquire the binary masks B, which were finally applied to every $l^{C}$ layer. Dynamically setting *p*, depending on the nature of each filter and the magnitude of the layer’s units, is justified; convolution filters are more sensitive to dropout compared to fully connected layers, as they directly interact with the input. Additionally, considering the spatial correlation of their units, applying dropout to the first convolution layers is expected to result in performance loss, as these layers are responsible for extracting the fundamental features of the input. Column 8 expresses the intervals of gradient masking; on some occasions, the binary masks were extracted after accumulating gradients for a number of epochs, in order to let the dropout mechanism have a broader knowledge of the impact of each unit on the training procedure. Finally, the 9th column holds the intervals of weight masking. Although the proposed method applies temporary dropout to the gradients of the hidden units, the binary masks can occur from the intensity of the gradients or a combination of the layer’s gradients and weight values.

On the CIFAR10 dataset, the proposed method outperformed the standard dropout architecture in most circumstances. Additionally, as seen in Table 1, the models that utilize the proposed dropout mechanism only need a few training epochs to score close to their best performances. In experiment 5, for a small performance trade-off (less than 1%), the model converged in just six epochs, which is a 168.4% reduction compared to the 70 epochs needed when training with standard dropout. As already discussed, intense selective dropout was only applied on the first fully connected layer, to the extreme of p=0.7, in experiments 3 and 4.

On the USPS dataset, our method outperformed the vanilla dropout architecture in all experiments, as seen in Table 2. Experiments 1 and 2 improved the network’s performance by 0.5%; in experiment 1, it performed its best in 62% fewer epochs than the vanilla version needed. In experiment 3, it needed fewer training steps to outperform the vanilla version by 0.25%, as its best performance reduced the required steps by 108.2%.

On Fashion-MNIST, the proposed method surpassed the standard dropout version by 2.04% (experiment 1) in 52.6% fewer epochs. In the third experimental setup, our method outperformed the baseline by 1.33% the fastest, in 23 epochs, or 70.4% fewer steps. All reported training procedures on the Fashion-MNIST dataset used weight masking in every epoch, as this setup was found to perform the best.

On STL-10, the best performing experimental setup achieved a 2.18% increase in performance, compared to the vanilla version, and only needed 22 epochs, a 127.2% reduction in convergence time. Setup 4 achieved a performance improvement of 0.71% in just 12 epochs, or 156.7% less epochs than the original dropout version. Weight masking was applied every epoch, and all setups accumulated gradients before freezing the dropout candidate parts.

Finally, on the SVHN dataset, the standard dropout version performed its best much quicker than on the previous datasets; however, the proposed method still outperformed it in terms of both accuracy and epochs needed. In our best experiment, 1, our algorithm surpassed the vanilla one’s performance by 0.26% in 34.5% fewer epochs. The fastest experiment for our method, 5, resulted in optimal performance almost identical to that of the vanilla version, being inferior by only 0.02%, but also faster by 109%.

## 5. Conclusions

In this paper, we proposed a novel algorithm for selectively disabling weight updating on parts of the network, based on both gradient and weight values of the respective network units. The proposed idea was tested in five well-known image classification datasets, yielding favorable performance results. Although the limited processing power restricted the architecture to an essential convolution network, the extended experiments have shown that this alternating scheme is able to match or surpass standard dropout performance in considerably fewer training steps. Convergence time is extremely important in practical machine learning applications; shorter network training times enable researchers to acquire knowledge quickly, and therefore conduct extended and more meaningful experiments. More importantly, faster production of models translates into additional ideas and methods for circumventing potential obstacles in research and integration.

## Figures and Tables

**Table 1:** Accuracy scores, parameter tuning, and convergence times for different experiments on the CIFAR10 dataset. The first row holds the best accuracy score for the vanilla dropout version and the epoch at which it was attained.

CIFAR10									
#	acc	epoch	conv1	conv2	conv3	conv4	fc1	int	wMask
v	72.8	70							
1	**73.26**	93	0.01	0.01	0.05	0.08	0.5	10	10
2	72.97	49	0.001	0.002	0.003	0.05	0.4	1	1
3	72.74	78	0.001	0.002	0.004	0.01	0.7	10	10
4	72.25	51	0.0	0.0	0.0	0.0	0.7	1	-
5	71.9	**6**	0.01	0.02	0.05	0.1	0.4	1	10

**Table 2:** Accuracy scores, parameter tuning, and convergence times for different experiments on the USPS dataset. The first row holds the best accuracy score for the vanilla dropout version and the epoch at which it was attained.

USPS									
#	acc	epoch	conv1	conv2	conv3	conv4	fc1	int	wMask
v	96.21	94							
1	**96.71**	49	0.0	0.0	0.0	0.0	0.7	1	-
2	**96.71**	85	0.01	0.01	0.02	0.03	0.3	1	4
3	96.46	**28**	0.002	0.002	0.003	0.05	0.2	1	1
4	96.41	92	0.001	0.001	0.01	0.2	0.5	5	1
5	96.41	53	0.001	0.001	0.002	0.01	0.1	1	20

**Table 3:** Accuracy scores, parameter tuning, and convergence times for different experiments on the fashion-MNIST dataset. The first row holds the best accuracy score for the vanilla dropout version and the epoch at which it was attained.

Fashion-MNIST									
#	acc	epoch	conv1	conv2	conv3	conv4	fc1	int	wMask
v	90.51	48							
1	**92.55**	28	0.0	0.002	0.007	0.01	0.5	1	1
2	92.26	35	0.008	0.008	0.008	0.008	0.8	1	1
3	91.84	**23**	0.005	0.01	0.05	0.1	0.5	1	1
4	91.44	34	0.005	0.005	0.01	0.02	0.5	1	1
5	91.36	43	0.01	0.01	0.01	0.2	0.5	1	1

**Table 4:** Accuracy scores, parameter tuning, and convergence times for different experiments on the STL-10 dataset. The first row holds the best accuracy score for the vanilla dropout version and the epoch at which it was attained.

STL-10									
#	acc	epoch	conv1	conv2	conv3	conv4	fc1	int	wMask
v	50.08	99							
1	**52.26**	22	0.001	0.001	0.002	0.05	0.2	5	1
2	51.76	100	0.001	0.002	0.01	0.01	0.1	2	1
3	50.86	28	0.1	0.1	0.15	0.2	0.4	5	1
4	50.79	**12**	0.02	0.02	0.1	0.2	0.4	5	1
5	50.56	48	0.001	0.001	0.002	0.01	0.3	5	5

**Table 5:** Accuracy scores, parameter tuning, and convergence times for different experiments on the SVHN dataset. The first row holds the best accuracy score for the vanilla dropout version and the epoch at which it was attained.

SVHN									
#	acc	epoch	conv1	conv2	conv3	conv4	fc1	int	wMask
v	90.19	17							
1	**90.45**	12	0.001	0.001	0.002	0.01	0.6	1	1
2	90.43	11	0.001	0.003	0.008	0.01	0.5	1	1
3	90.36	12	0.001	0.001	0.01	0.02	0.5	1	1
4	90.25	26	0.001	0.001	0.002	0.005	0.6	1	1
5	90.17	**5**	0.001	0.003	0.008	0.01	0.3	1	2

## Data Availability

Not applicable.
